# Osteo Sarcoma

**Published:** 1877-07

**Authors:** F. Peabody


### OSTEO SARCOMA.

BY F. PEABODY D. D. S.

November the 25th, Dr. G———, brought his daughter, a beautiful girl of eighteen, to have me examine her mouth, and to ascertain the cause of a slight enlargement on the right side. On examination I found, between the inferior cuspidati and first bicuspid, which stood about an eighth of an inch apart, a slight enlargement.

On the lingual surface of the maxillary was an enlargement about three quarters of an inch in diameter, extending over the cuspidati and first bicuspid, the centre being opposite the space between the teeth. It was puffed out about a quarter of an inch and almost black in color. On the buccal portion the enlargement was not as great nor quite so dark in color, but had an unpleasant livid appearance. The young lady had experienced no pain or inconvenience from it, and had not mentioned it to her parents, though she was aware of it in May, of the same year. It was only on her mother noticing that her cheek appeared to be slightly swolen, that she spoke of it. This was a few days before I saw her. I found, while on the lingual side, the bone itself appeared to be puffed out; on the buccal side the bone where the parts were enlarged was absent. The gum over the absent bone was livid and exceedingly elastic, and indicated deep seated fluctuation. Having concluded to open it, I plunged a lancet in, but got nothing save a quantity of dark, almost black blood. A small probe introduced passed through the maxillary nearly to the lingual surface. The plate of bone there not being over one sixteenth of an inch in thickness. Anteriorly the probe passed almost to the cuspidati and posteriorly passed the first bicuspid, the root of which on its anterior approximal, buccal and lingual sides was exposed to the cavity. No portion of the bone could be felt; on all sides of the cavity was a soft fungus growth of flesh. All the teeth were sound and in place excepting the first bicuspid, which was sound but leaned inward until the inner cusp was on a line with the gum. The teeth were also perfectly firm. I advised the removal of the first bicuspid, but as the young lady was somewhat nervous I did not press the matter at that time. Injecting the cavity with Elix. Vit. and putting a tent into the opening that had been made with the lancet, I dismissed her, and requested her to return in three days.

On her return I found the discoloration on the lingual surface almost gone, the parts presented almost a normal appearance, with the exception of the enlargement, but the buccal portion was not improved. It was not quite as dark but more livid. I found the first bicuspid somewhat loose, and again advised its removal, but the patient objected so strongly I consented to its remaining until the next sitting. I again injected with Elix. Vit. dismissed for three days. At the next sitting, finding no improvement, I insisted on removing the bicuspid, which after some persuasion she consented to, though quite loose it was much harder to extract than I had anticipated. Much dark blood came from the socket. I found on the anterior, inferior half of the root, a peculiar granular deposit of what appeared to be flesh, under a powerful glass it seemed to be a series of globules connected together, and covered by a membrane which was intact; that is, it evidently was not torn from any of the fungi which covered the interior of the cavity. I treated the socket with iodine and creosote, and requested the patient to ask her father to come with her next time.

When she returned there was no improvement, and having shown her father the tooth, I expressed my fears as to the serious nature of the tumor, and advised its immediate removal by an operation. While pondering on the subject the idea occured to him to use electricity, and his son reported to me the next day that on the first application the gums had shrunk, or shriveled up almost to their natural condition, and the parts had resumed their normal color. I of course expressed my gratification at the report, but could not believe that electricity could effect a cure of a tumor of such an alarming character.

In a few days I saw it again, it looked no better than when I last saw it, and there was a marked malignant appearance that caused me again to suggest to her father that he should lose no time in having it operated on. Being the only young daughter he had, and one who bid fair to make an exceedingly pretty woman, he naturally felt loth to proceed to such an extremity until all other means had been exhausted. The tumor now began to be somewhat painful and quite sensitive to the touch. He sent for an electrician and tried electrolysis. Passing two needles with gold points through the tumor, one from the lingual side, the other from the buccal until the points nearly met, the battery was applied. The shock was exceedingly painful, and the current could only be kept up for about ten minutes. Some ten days passed when it was applied again. In about ten days more no improvement having taken place it was concluded to make the operation.

The patient having been put under a mixture of chloroform, ether and alcohol, the tumor was probed to discover its dimensions; the probe did not pass beyond the septum of the cuspidati tooth, which on its posterior approximal, and lingual sides seemed to be intact, posteriorly it passed beyond the second bicuspid. It being desirable, if possible, to save the cuspidati.

Dr. Hollaway, the operator, passed the saw down close to that tooth, and on the anterior side of the first molar, (the second bicuspid having been previously extracted,) and after some difficulty in applying the cutting forceps the bone was removed. Introducing my finger into the cavity to ascertain that the disease had not passed beyond the cuspidati, to my surprise it slipped in half an inch on the labial side of that tooth into a mass of soft fungi. I had not anticipated this as I was more inclined to believe that the tumor had burrowed posteriorly. On account of the thickness of the parts the probe had not reached this point, and the gum over it showed no indication of the trouble beneath. The cuspidati was now removed, also the lateral incisor, and all the diseased mass with the bone cut away. It was also deemed advisable to remove the first molar, (as the tumor had extended quite up to it,) and to cut away the process and bone. After the tumor had been removed, the bottom of the bone where the fungi had rested was as smooth as though it had been polished, and the thickness from there to the base of the maxillary was not even a quarter of an inch. Great care was exercised in removing every trace of the disease, and the operation was a very thorough one. At the first cut the blood flowed profusely, but little hemorrhage followed however, and caused no inconvenience. In ten days the patient was out of bed, and in two weeks out in the street. The parts granulated very rapidly and kindly, and she experienced but little pain after the operation.

The report made by Dr. Marvin, microscopist, is as follows:

"The tumor removed from the mouth of Miss G———, has been examined with the following results:"

The mass was moderately firm and resistant under the knife. No appreciable quantity of juice exuding. Color dark reddish, slightly mottled. By moistening the cut surface and examining with microscope a great number of cells appeared, varying in size and shape. The prevailing kind were round oval irregularly shaped, tailed cells, containing from one to a dozen large, oval nucleated nuclei, some oat shaped cells with same kind of nuclei, also a large number of free nucleated nuclei; sections of the mass revealed same kind of cells. The proper substance of the gums being entirely replaced by these cells in various stages of development. The mass presents in an unusual degree all the characteristics of the myeloid tumor (of Paget) or sarcoma colossal (of Virchow) or fibroplastic (of Lebert.) This kind of tumor is frequent in young persons especially liable to attack the jaws. It is classed among the benign tumors by all the authorities.

